# Sensitivity analysis on natural convective trapezoidal cavity containing hybrid nanofluid with magnetic effect: Numerical and statistical approach

**DOI:** 10.1016/j.heliyon.2024.e41508

**Published:** 2024-12-26

**Authors:** Sweety Khatun, Rupa Kundu, Saiful Islam, Ritu Aktary, Dipankar Kumar

**Affiliations:** Department of Mathematics, Bangabandhu Sheikh Mujibur Rahman Science and Technology University, Gopalganj, 8100, Bangladesh

**Keywords:** Natural convection, Magnetohydrodynamic, Hybrid nanofluid, Sensitivity analysis, Nusselt number

## Abstract

The numerical analysis examines the attributes of magnetohydrodynamic natural convection in a closed cavity including a circular hollow. Because mono and hybrid nanofluids have many applications in thermal engineering and manufacturing, hybrid nanofluids are utilized as the substance within the entire domain. The investigation centers on a closed, trapezoidal-shaped hollow with a heated surface ring. The inclined vertical walls are treated as cold exteriors, while the others are insulated. Another factor is taking a magnetic field horizontally to the cavity. The entire cavity contains a hybrid nanofluid, an amalgamation of *SiO*_*2*_ and *Ag* nanoparticles with water. The associated governing equations are simulated via the finite element method. Exploiting streamlines, isotherms, and line graphs, the results are physically explained for a range of values of Rayleigh number (10^3^ ≤ *Ra* ≤ 10^6^), nanoparticle volume fraction (0 ≤ *ϕ* ≤ 0.1), and Hartmann number (0 ≤ *Ha* ≤ 100). The RSM is used to display 2*D* and 3*D* effects of significant factors on response function. A best-fitted correlation is built up to examine the rate of sensitivity. It is found that incorporating hybrid nanoparticles and intensifying the Rayleigh number directs to the thermal actuation of hybrid nanofluid. In the event of a growing magnetic impact, reverse behaviors should be noted. The water's ability to transmit heat increases to 11.29 % when the *Ag-SiO*_*2*_*-H*_*2*_*O* hybrid nanofluid is used. The imposition of a magnetic field (*Ha* = 25), the rate of heat transmission lessens to 2.4 %. The *Ra* has positive sensitivity whereas *Ha* shows inverse behavior. Lastly, the study's findings might guide designing a successful natural convective mechanical device.

## 1 Introduction

Convective heat transfer is relevant to multi-engineering society because it has widespread use such as on heat exchangers, solar collectors, home ventilation systems, electrical equipment, petroleum production, and so on. It has also played a significant role in our daily lives. Free convection is the term for natural convection, which happens simultaneously due to temperature differences. Besides the impulsive nature that makes natural convection desirable. Additionally, it gets rid of the risks of mechanical failure that come with forced convection systems. The elimination of extra mechanical components leads to substantial financial savings in addition to decreases in overall size, noise level, and electromagnetic interference limitations which are often essential requirements in the specifications of industrial equipment. A few reimbursements like these highlight the increasing focus on applying this heat transfer technology, particularly for equipment placed in tight places (or cavities), which is a common occurrence in many technical applications. Power plants, geothermal reservoirs, mechanical engineering, geophysics, electrical technology, and heat exchanger design are among the domains where cooling systems and heat transmission in enclosed spaces are major concerns. Scientists and researchers already conducted several investigations to enhance the enclosed cavity's cooling system and heat exchanger by gaining a better comprehension of the natural convective heat transfer mechanism [[1], [2], [3], [4], [5]]. Fontana et al. [6] studied free convection in a trapezoidal inclusion having upper and bottom walls taken as adiabatic, and it was supposed that upright walls were insulated. The influence of the inclination angle of plate-fin heat sinks was investigated by Tari et al. [7] where numerous correlations were built for different cases of inclinations. In a trapezoidal cavity, a 3D investigation was conducted by Benhamou et al. [8] using the lattice Boltzmann method (LBM). They concluded that when the Rayleigh number rises, entropy formation via viscosity takes precedence over entropy production from the exchange of heat. Kumar et al. [9] undertook a computational study, as well as an experimental inquiry, into natural air convection in a partially uncovered square container with two interior bases of heat. To examine free convection behavior, Öztop et al. [10] created a fluid model in a square cavity with two semi-circular heaters.

Once again, a fluid that includes nanoparticles, such as *Co*, *Zn*, *Ag*, *Al*_*2*_*O*_*3*_, *TiO*_*2*_, and *Fe*_*3*_*O*_*4*_, which are particles the size of nanometers, is referred to as a nanofluid. Examples of such fluids, known as base fluids, are water, engine oil, kerosene, and others. Many industrial and technological fields, including healthcare, solar energy, heat exchangers, and nuclear reactor applications, have been using these nanofluids which have many promising advantages as noble fluids for a long time [[11], [12], [13], [14], [15], [16]]. To increase the ordinary fluids' thermal conductivity, American scientist S.U.S. Choi [17] of Argonne National Laboratory developed the term "nanofluids" in 1995. He did this by suspending solid nanoparticles into base fluids. Subsequent research revealed that the addition of nanoparticles to regular fluids in a range of settings enhanced thermal conductivity, which sped up the transmission of heat. However, studies conducted by several experts have shown that nanofluids have a considerably larger rise in heat transport than traditional fluids. It suggests that there are other elements causing nanofluids to have strong heat transmission capabilities [18]. Maximum properties of nanofluids are affected by several factors, including temperature, concentration, particle motion, and the kind and size of the particles. Metal oxide nanoparticles (like *TiO*_*2*_, *CuO*, *Al*_*2*_*O*_*3*_, etc.) provide unique properties and stability, but metal-type nanoparticles (like *Cu*, *Al*, *Ag*, *Ti,* and *Au*, etc.) increase heat conductivity but are thermally unstable and prone to changes. Hybrid nanofluids, which blend metallic and metallic oxide solid nanoparticles into base fluids, can produce advantageous results regarding physical properties, stability, and chemical reactivity [[19], [20], [21]]. The enhancement of heat transfer in various physical structures with different boundary conditions and numerous geometries has been studied numerically in the past few years using hybrid nanofluids. Tayebi et al. [22] studied about numerical development of free convection for a hybrid nanofluid in an annulus that was both elliptical and cylindrical. Khadim et al. [23] utilized a hybrid nanofluid to study the effect of adding *Cu* and *Al*_*2*_*O*_*3*_ nanoparticles into water in a wavy porous arena. Their investigation indicates that higher thermohydrodynamic efficiency was obtained when a hybrid fluid is used as an alternative to a traditional nanofluid. Another investigation on *Cu-Al*_*2*_*O*_*3*_*-H*_*2*_*O* nanofluid was completed by Suresh et al. [24] and concluded that the heat rate of heat transfer due to convection (Nu_av_) was developed by 8.02 % compared to pure water. In comparison to conventional water, the Nu_av_ was observed to be approximately 30–35 % higher when Balla et al. [25] examined the flow of a hybrid nanofluid based on *CuO* and *Cu* nanoparticles in a round conductor. Megatif et al. [26] discovered a significant augmentation of Nu_av_ and thermal conductivity factor for a hybrid nanofluid (*TiO*_*2*_*-CNT-H*_*2*_*O*). Similarly, Tayebi et al. [27] evaluated natural convection within an annular cavity that was filled by *Cu-Al*_*2*_*O*_*3*_*-H*_*2*_*O*. They concluded that Nu_av_ and entropy generation were developed when *Ra* and nanoparticle concentration were increased. Mohebbi et al. [28] explored the processes of natural convection in a T-type enclosure that had a section covered by a porous medium and a hybrid fluid. Their results showed that *Ra* number and *ϕ* increased the magnitude of Nu_av_.

Additionally, in the existence of a magnetic field, the temperature and flow fields are governed by the combined action of the Lorentz and buoyancy forces. In the case of electrically conducting fluid flow, the magnetohydrodynamic (MHD) force is likewise dynamic. Numerous investigations have been completed on MHD flow because of its importance in real life such as in metal casting, fusion reactors, extraction of geothermal energy, growth of liquid crystal, polymer manufacturing, and metallurgy [[29], [30], [31], [32]]. Mebarek-Oudina et al. [33] investigated the efficiency of thermal systems, incorporating magnetic fields with entropy generation, using an MHD hybrid nanofluid within a porous elliptical-shaped chamber. Selimefendigil et al. [34] analyzed natural convection in a 3*D* corrugated trapezoidal space employing a nanofluid (*CuO-H*_*2*_*O*). They found that when MHD force was applied, the local and average Nusselt numbers climbed up due to the improvement of *Ra* and *ϕ*. These heat transmission losses also worsen with an increase in *Ha* number. To study the belongings of radiation over a porous sheet employing *TiO*_*2*_*-Ag-H*_*2*_*O* nanofluid, Mahesh et al. [35] recently carried out another experiment on MHD. In a permeable curved cavity, Mandal et al. [36] examined MHD free convective thermal carriage of hybrid nanofluid, wherever bolstering the modified-*Ra* increased the quantity of heat transmission. Ahmed et al. [37] investigated the effects of *Al*_*2*_*O*_*3*_*-Cu-H*_*2*_*O*-based radiative MHD natural convection in wave-patterned porous structures.

According to the above-mentioned literature review, MHD natural convection is of great interest to academic research due to its novel implications across a wide range of engineering disciplines. Despite countless research on multiple closed cavities over time to explore the performance of MHD-natural convection, only a few studies on trapezoidal attachments using distinct mono and hybrid nanofluids were completed at various times [6,33,34]. Again, in multiple studies, the response surface methodology (RSM) was used to depict response surfaces and explore the sensitivity of the response function to independent factors [38,39]. FEM, which is so much more suitable for complex-shaped geometry rather than any other numerical method, is applied to get a numerical solution. However, in this study, the RSM and FEM are applied to a trapezoidal cavity with a circular hot surface that contains an *Ag*-*SiO*_*2*_-*H*_*2*_*O* hybrid nanofluid. Researchers have previously studied the individual (*Ag* and *SiO*_*2*_) use of these two nanoparticles, but not their combined use. This is the main rationale behind the selection of *Ag* and *SiO*_*2*_. Applying RSM generates a best-fitted relationship between the response function and the relevant parameters. The article also explains the 2*D* and 3*D* response functions for *Ra*, *Ha*, and *ϕ*. That is, the consequence of a hybrid nanofluid combining *Ag* and *SiO*_*2*_ nanoparticles with water in a natural convective trapezoidal cavity is investigated for the first time.

## 2 Physical descriptions and mathematical model

**Fig. 1:**
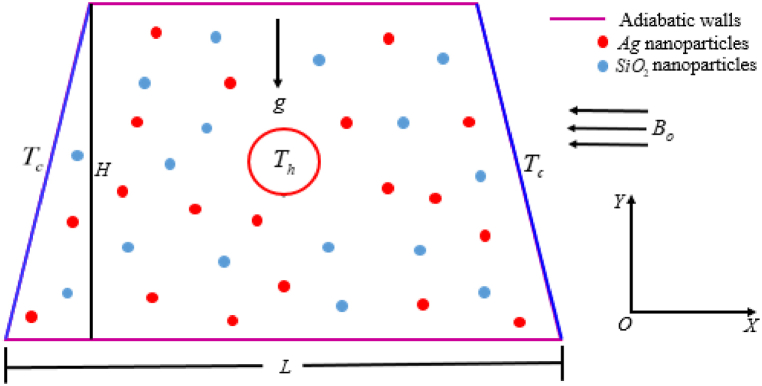
Proposed model's schematic diagram.

In this computational analysis, water (*H*_*2*_*O*) is used as base fluid, and silver (*Ag*) and silicon dioxide (*SiO*_*2*_) nanoparticles are confined within a trapezoidal-shaped enclosure to make a hybrid nanofluid. This fluid, which includes the effects of a magnetic field, is a stable, incompressible Newtonian fluid. A hot surface (*T*_*h*_) is defined as a cylindrical pipe (cross-section) with a diameter of 0.1*L* and a center of (*X*, *Y*) = (0.5, 0.3). Two parallel walls are reserved for thermal insulation, but two inclined walls are regarded as cooler surfaces (*T*_*c*_). The physical configuration of this model is depicted in Fig. 1. The gravitational acceleration (*g*) acts along the negative path of the *Y*-axis. In addition to the enclosure, the magnetic field *B*_*0*_ is incorporated from right to left. Both the neighboring sides and the homogenous forms of the *SiO*_*2*_ and *Ag* nanoparticles are considered non-slip. In this instance, Table 1 describes the thermo-physical characteristics of the solid nanoparticles and base fluid. A two-dimensional coordinate system where the vertically inclined walls are represented by the *Y*-axis and the nethermost by the *X*-axis. Actually, in real life, there are numerous industrial architecture as trapezoidal shapes that contain pipes containing numerous fluids. As a result, such a type of trapezoidal domain is selected to analyze the fluid flow and heat transfer performance.

**Table 1:** Properties of base fluid and solid nanoparticles [33,40].

	c_p_	ρ	κ	β	σ	μ
*H*_*2*_*O*	4179	997.1	0.613	21 × 10^−5^	5.5 × 10^−6^	8.91 × 10^−6^
*Ag*	235	10,500	429	5.5 × 10^−4^	8.1 × 10^−4^	–
*SiO*_*2*_	745	2200	1.4	42.7 × 10^−5^	10^−12^	–

Here, *c*_*p*,_ *ρ*, *κ, β, σ* and *μ* represent specific heat at constant pressure, density, thermal conductivity, coefficient of thermal expansion, electric conductivity, and coefficient of viscosity, respectively. To form a mathematical model for this MHD natural convective model using hybrid nanofluid in a trapezoidal cavity, the following equations are taken [41]:

(1) $$\frac{\partial u}{\partial x}+\frac{\partial v}{\partial y}=0$$

(2a) $$\frac{\partial u}{\partial x}+\frac{\partial u}{\partial y}=-\frac{1}{\rho_{hnf}}\left(\frac{\partial p}{\partial x}\right)+\frac{\mu_{hnf}}{\rho_{hnf}}\left(\frac{\partial^{2}u}{\partial x^{2}}+\frac{\partial^{2}u}{\partial y^{2}}\right)$$

(2b) $$\frac{\partial v}{\partial x}+\frac{\partial v}{\partial y}=-\frac{1}{\rho_{hnf}}\left(\frac{\partial p}{\partial y}\right)+\frac{\mu_{hnf}}{\rho_{hnf}}\left(\frac{\partial^{2}v}{\partial x^{2}}+\frac{\partial^{2}v}{\partial y^{2}}\right)+\frac{g{\left(\rho \beta \right)}_{hnf}}{\rho_{hnf}}\left(T-T_{c}\right)-\frac{\sigma_{hnf}B_{0}^{2}}{\rho_{hnf}}v$$

(3) $$\frac{\partial T}{\partial x}+\frac{\partial T}{\partial y}=\frac{\kappa_{hnf}}{{\left(\rho c_{p}\right)}_{hnf}}\left(\frac{\partial^{2}T}{\partial x^{2}}+\frac{\partial^{2}T}{\partial y^{2}}\right)$$

where ρhnf,μhnf,(ρβ),hnfσhnfandkhnf stand for the hybrid nanofluids' density, dynamic viscosity, thermal expansion coefficient, electrical conductivity, and thermal conductivity, respectively. Moreover, the following are the associated dimensional boundary conditions [14,39]:

(4) $$\begin{array}{l} T=T_{h},u=0,v=0,\text{on}\;\text{circular}\;\text{surface}\\ T=T_{c},u=0,v=0,\text{on}\;\text{vertical}\;\text{inclined}\;\text{walls}\\ \frac{\partial T}{\partial n}=0,u=v=0,\text{on}\;\text{all}\;\text{others}\;\text{walls} \end{array}\}$$

Here, *n* is the perpendicular unit vector along the *XY* plane. The relationships that are taken into consideration between base fluid (*H*_*2*_*O*) and nanoparticles (*Ag* and *SiO*_*2*_) are explained in this section. In reality, base fluid and nanoparticles influence the characteristics of nanofluids. Thus, Table 2 correlations are used to compute nanofluid characteristics.

**Table 2:** Applied relationships of hybrid nanofluid [33,39,43].

Properties:		Applied Correlations:
Concentration of nanoparticles	:	$\phi =\phi_{Ag}+\phi_{SiO_{2}}$
Nanofluid's density	:	$\rho_{hnf}=\left(1-\phi \right)\rho_{bf}+\phi \rho_{sp}$ where $\phi \rho_{sp}=\phi_{Ag}\;\rho_{Ag}+\phi_{SiO_{2}}\rho_{SiO_{2}}$
Dynamic viscosity	:	$\mu_{hnf}=\mu_{bf}\;\left(1+2.5\phi +6.5\phi^{2}\right)$
Specific heat capacity	:	${\left(\rho c_{p}\right)}_{hnf}=\left(1-\phi \right){\left(\rho c_{p}\right)}_{bf}+\phi {\left(\rho c_{p}\right)}_{sp}$ where $\phi {\left(\rho c_{p}\right)}_{sp}=\phi_{Ag}{\left(\rho c_{p}\right)}_{Ag}+\phi_{SiO_{2}}{\left(\rho c_{p}\right)}_{SiO_{2}}$
Thermal diffusivity	:	$\alpha_{hnf}=\frac{\kappa_{hnf}}{{\left(\rho c_{p}\right)}_{hnf}}$
Thermal conductivity	:	$k_{hnf}=k_{bf}\left\{\frac{k_{sp}+2k_{bf}-2\phi \left(k_{bf}-k_{sp}\right)}{k_{sp}+2k_{bf}+\phi \left(k_{bf}-k_{sp}\right)}\right\}$ where $\phi \kappa_{sp}=\phi_{Ag}k_{Ag}+\phi_{SiO_{2}}\;\kappa_{SiO_{2}}$
Thermal expansion coefficient	:	${\left(\rho \beta \right)}_{hnf}=\left(1-\phi \right){\left(\rho \beta \right)}_{bf}+\phi {\left(\rho \beta \right)}_{sp}$ where $\phi {\left(\rho \beta \right)}_{sp}=\phi_{Ag}{\left(\rho \beta \right)}_{Ag}+\phi_{SiO_{2}}{\left(\rho \beta \right)}_{SiO_{2}}$
Electrical conductivity	:	$\sigma_{hnf}=\sigma_{bf}\left[1+\frac{3\phi \left(\frac{\sigma_{sp}}{\sigma_{bf}}-1\right)}{\left(\frac{\sigma_{sp}}{\sigma_{bf}}+2\right)-\phi \left(\frac{\sigma_{sp}}{\sigma_{bf}}-1\right)}\right]$ where $\phi \;\sigma_{sp}=\phi_{Ag}\sigma_{Ag}+\phi_{SiO_{2}}\phi_{SiO_{2}}$

After completing the dimensionless calculation, the governing equations (1)–(3)become:

(5) $$\frac{\partial U}{\partial X}+\frac{\partial V}{\partial Y}=0$$

(6) $$U\frac{\partial U}{\partial X}+V\frac{\partial U}{\partial Y}=-\left(\frac{\rho_{bf}}{\rho_{hnf}}\right)\frac{\partial P}{\partial X}+\left(\frac{\mu_{hnf}}{\mu_{bf}}.\frac{\rho_{bf}}{\rho_{hnf}}\right)\mathrm{Pr}\left(\nabla^{2}U\right)$$

(7) $$U\frac{\partial V}{\partial X}+V\frac{\partial V}{\partial Y}=-\left(\frac{\rho_{bf}}{\rho_{hnf}}\right)\frac{\partial P}{\partial Y}+\left(\frac{\mu_{hnf}}{\mu_{bf}}.\frac{\rho_{bf}}{\rho_{hnf}}\right)\mathrm{Pr}\left(\nabla^{2}V\right)+\frac{{\left(\rho \beta \right)}_{hnf}}{\rho_{hnf}\beta_{bf}}Ra\;\mathrm{Pr}\;\theta -\left(\frac{\rho_{bf}}{\rho_{hnf}}.\frac{\sigma_{hnf}}{\sigma_{bf}}\right)\mathrm{Pr}\;Ha^{2}\;V$$

(8) $$U\frac{\partial \theta }{\partial X}+V\frac{\partial \theta }{\partial Y}=\left(\frac{\alpha_{hnf}}{\alpha_{bf}}\right)\nabla^{2}\theta$$

where the applied dimensionless quantities are [14,39]:

(9) $$\begin{array}{l} X=\frac{x}{L},Y=\frac{y}{L},U=\frac{uL}{\alpha_{bf}},V=\frac{\upsilon L}{\alpha_{bf}},P=\frac{pL^{2}}{\rho_{bf}\;\alpha_{bf}^{2}},\theta =\frac{T-T_{c}}{T_{h}-T_{c}}\text{,}\\ \text{Ra}=\frac{g\;\beta_{bf}\left(T_{h}-T_{c}\right)L^{3}}{\upsilon_{bf}\alpha_{bf}},\mathrm{Pr}=\frac{\upsilon_{bf}}{\alpha_{bf}},\text{and}\;\text{Ha}=B_{0}L\sqrt{\frac{\sigma_{bf}}{\mu_{bf}}} \end{array}\}$$

Also, the dimension-free boundary conditions look as:

(10) $$\begin{array}{l} \theta =1,U=V=0,\text{on}\;\text{circular}\;\text{surface}\\ \theta =0,U=V=0,\text{on}\;\text{vertical}\;\text{inclined}\;\text{boundaries}\\ \frac{\partial \theta }{\partial N}=0,U=V=0,\text{on}\;\text{all}\;\text{others}\;\text{walls} \end{array}\}$$

Where *N* is the upright unit vector on the *XY* plane. In addition, the mean rate of heat transfer [14,39] from the heated circular exterior can be computed using equation (11).

(11) $${\text{Nu}}_{\text{av}}=-\left(\frac{k_{hnf}}{k_{bf}}\right)\int_{\text{HS}}\frac{\partial \theta }{\partial N}\;dS$$

Where HS denotes the heated surface. Also, the stream function is formulated as: $U=\frac{\partial \psi }{\partial Y},V=-\frac{\partial \psi }{\partial X}$. The outside walls are entirely coated with non-slip material. Furthermore, the vorticity equation is: $\frac{\partial^{2}\psi }{\partial X^{2}}+\frac{\partial^{2}\psi }{\partial Y^{2}}=-\left(\frac{\partial V}{\partial X}-\frac{\partial U}{\partial Y}\right)=-$ Ω, where the vorticity vector is denoted by Ω, and *U* and *V* express the velocities along the *X* and *Y* axes, respectively.

## 3 Numerical simulation

### 3.1 Solution methodology

Using conditions (9), the Galerkin weighted residual finite element method (FEM) is utilized for solving the non-dimensional governing equations (5), (6), (7), (8) for this model. Temperature and velocity may be calculated using quadratic interpolation techniques since the entire fluid region is separated into discrete triangular-form elements with six nodes. The pressure gradient is also estimated using the linear interpolation approach. The penalty finite element method is used to solve equation (6) through (8), where *P* is replaced by the penalty constant γ. Thus, equation (5) can be written as follows:

(12) $$P=-\gamma \left(\frac{\partial U}{\partial X}+\frac{\partial V}{\partial Y}\right)$$

A greater value of γ automatically satisfies the continuity equation. Equations (6), (7) are reduced to:

(13) $$U\frac{\partial U}{\partial X}+V\frac{\partial U}{\partial Y}=\gamma \frac{\rho_{bf}}{\rho_{hnf}}\frac{\partial }{\partial X}\left(\frac{\partial U}{\partial X}+\frac{\partial V}{\partial Y}\right)+\frac{\mu_{hnf}}{\mu_{bf}}\frac{\rho_{bf}}{\rho_{hnf}}\mathrm{Pr}\left(\frac{\partial^{2}U}{\partial X^{2}}+\frac{\partial^{2}U}{\partial Y^{2}}\right)$$

(14) $$U\frac{\partial V}{\partial X}+V\frac{\partial V}{\partial Y}=\gamma \frac{\rho_{bf}}{\rho_{hnf}}\frac{\partial }{\partial Y}\left(\frac{\partial U}{\partial X}+\frac{\partial V}{\partial Y}\right)+\frac{\mu_{hnf}}{\mu_{bf}}\frac{\rho_{bf}}{\rho_{hnf}}\mathrm{Pr}\left(\frac{\partial^{2}V}{\partial X^{2}}+\frac{\partial^{2}V}{\partial Y^{2}}\right)+\frac{{\left(\rho \beta \right)}_{hnf}}{\rho_{hnf}\beta_{bf}}Ra.\mathrm{Pr}.\theta -\frac{\rho_{bf}\sigma_{hnf}}{\rho_{hnf}\sigma_{bf}}\mathrm{Pr}\;Ha^{2}V$$

The temperature (θ) and velocity (U, V) components are increased using basis sets ${\left\{\phi_{r}\right\}}_{r=1}^{N}$ as follows:

(15) $$U\approx \sum_{s=1}^{N}U_{s}\phi_{s}\left(X,Y\right),V\approx \sum_{s=1}^{N}V_{s}\phi_{s}\left(X,Y\right),\text{and}\;\theta \approx \sum_{s=1}^{N}\theta_{s}\phi_{s}\left(X,Y\right)\}$$

Next, the Galerkin FEM is used to get the subscript non-linear residual equations for equations (8), (13), (14) at nodes inside the domain Ω:

(16) $$Ri^{\left(1\right)}=\sum_{s=1}^{N}\theta_{s}\int_{\Omega }\left[\left(\sum_{s=1}^{N}U_{s}\phi_{s}\right)\frac{\partial \phi_{s}}{\partial X}+\left(\sum_{s=1}^{N}V_{s}\phi_{s}\right)\frac{\partial \phi_{s}}{\partial Y}\right]\phi_{i}dXdY-\frac{\alpha_{hnf}}{\alpha_{bf}}\sum_{s=1}^{N}\theta_{s}\int_{\Omega }\left(\frac{\partial \phi_{i}}{\partial X}\frac{\partial \phi_{s}}{\partial X}+\frac{\partial \phi_{i}}{\partial Y}\frac{\partial \phi_{s}}{\partial Y}\right)dXdY$$

(17) $$Ri^{\left(2\right)}=\sum_{s=1}^{N}U_{s}\int_{\Omega }\left[\left(\sum_{s=1}^{N}U_{s}\phi_{s}\right)\frac{\partial \phi_{s}}{\partial X}+\left(\sum_{s=1}^{N}V_{s}\phi_{s}\right)\frac{\partial \phi_{s}}{\partial Y}\right]\phi_{i}dXdY-\gamma \frac{\rho_{bf}}{\rho_{hnf}}\left[\sum_{s=1}^{N}U_{s}\int_{\Omega }\left(\frac{\partial \phi_{i}}{\partial X}\frac{\partial \phi_{s}}{\partial X}\right)dXdY+\sum_{s=1}^{N}V_{s}\int_{\Omega }\left(\frac{\partial \phi_{i}}{\partial X}\frac{\partial \phi_{s}}{\partial Y}\right)dXdY\right]-\frac{\mu_{hnf}}{\mu_{bf}}\frac{\rho_{bf}}{\rho_{hnf}}\mathrm{Pr}\sum_{s=1}^{N}U_{s}\int_{\Omega }\left(\frac{\partial \phi_{i}}{\partial X}\frac{\partial \phi_{s}}{\partial X}+\frac{\partial \phi_{i}}{\partial Y}\frac{\partial \phi_{s}}{\partial Y}\right)dXdY$$

(18) $$Ri^{\left(3\right)}=\sum_{s=1}^{N}V_{s}\int_{\Omega }\left[\left(\sum_{s=1}^{N}U_{s}\phi_{s}\right)\frac{\partial \phi_{s}}{\partial X}+\left(\sum_{s=1}^{N}V_{s}\phi_{s}\right)\frac{\partial \phi_{s}}{\partial Y}\right]\phi_{i}dXdY-\gamma \frac{\rho_{bf}}{\rho_{hnf}}\left[\sum_{s=1}^{N}U_{s}\int_{\Omega }\left(\frac{\partial \phi_{i}}{\partial Y}\frac{\partial \phi_{s}}{\partial X}\right)dXdY+\sum_{s=1}^{N}V_{s}\int_{\Omega }\left(\frac{\partial \phi_{i}}{\partial Y}\frac{\partial \phi_{s}}{\partial Y}\right)dXdY\right]-\frac{\mu_{hnf}}{\mu_{bf}}\frac{\rho_{bf}}{\rho_{hnf}}\mathrm{Pr}\sum_{s=1}^{N}V_{s}\int_{\Omega }\left(\frac{\partial \phi_{i}}{\partial X}\frac{\partial \phi_{s}}{\partial X}+\frac{\partial \phi_{i}}{\partial Y}\frac{\partial \phi_{s}}{\partial Y}\right)dXdY-\frac{{\left(\rho \beta \right)}_{hnf}}{\rho_{hnf}\beta_{bf}}Ra\;\mathrm{Pr}\int_{\Omega }\sum_{s=1}^{N}\left(\theta_{s}\phi_{s}\right)\phi_{i}dXdY+\frac{\rho_{bf}\sigma_{hnf}}{\rho_{hnf}\sigma_{bf}}\mathrm{Pr}\;Ha^{2}\int_{\Omega }\sum_{s=1}^{N}\left(V_{s}\phi_{s}\right)\phi_{i}dXdY$$

**Fig. 2:**
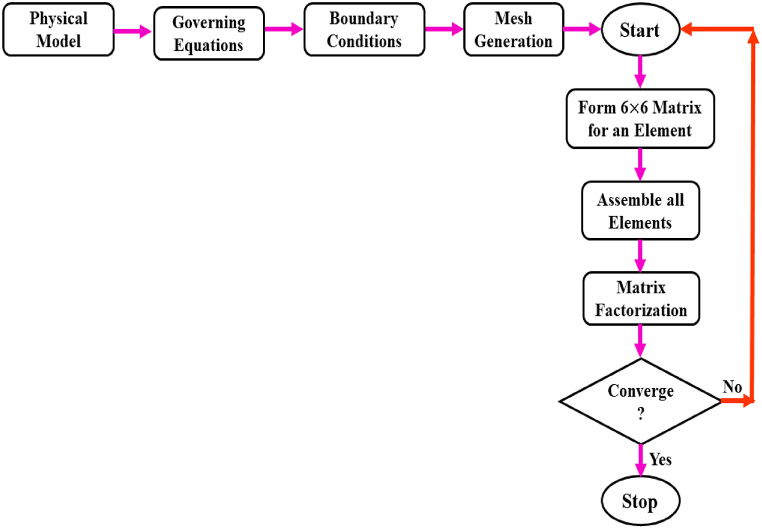
The computational procedure for FEM.

These governing equations can be solved in MATLAB by using the Newton-Raphson iteration technique, which yields a communal of global nonlinear algebraic equations. For every variable with less than δ, the weighted residual approach's convergence criterion is established so that $\left|\psi^{i+1}-\psi^{i}\right|<\delta =10^{-5}$ , where the iteration magnitude is indicated by ψ(U,V,θ), and i+1andi are two successive repetitions [42,44,45]. provided an explanation of the FEM process in its entirety. Fig. 2 depicts a broad flowchart of this computational method.

### 3.2 Grid sensitivity analysis

**Fig. 3:**
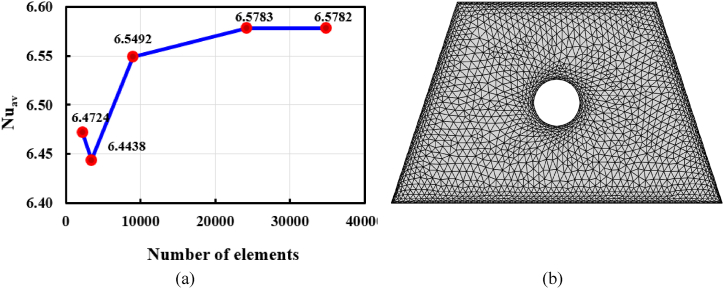
(a) Grid sensitivity test using element size and Nu_av_, and (b) discretization process.

To achieve the perfect number of meshing elements for this EFM, an analysis of grid size is presented by keeping *Ra* = 10^4^, *Ha* = 10, and Pr = 6.83. Also, the nanoparticle volume fraction (*ϕ*) of both *Ag* and *SiO*_*2*_ nanoparticles is 0.02. Again, as for the mesh construction activity, the magnitude of Nu_av_ is selected to do this analysis. There are five different sizes of triangle elements in the domain: 2225, 3416, 8992, 24235, and 34809, in that order. Fig. 3(a) shows a line graph that illustrates Nu_av_'s grid sensitivity test for several meshing types. The amount of Nu_av_ for 24235 elements is similar to the subsequent number of meshing types, despite some changes for the first two meshing types (2225 and 3416) shown in Table 3 and Fig. 3(a). As a result, 24235 elements are used for final discretization to complete this study. Fig. 3(b) represents the meshing generated figure of the given distribution.

**Table 3:** Magnitude of Nu_av_ with different sizes of elements.

**Elements**	2225	3416	8992	24235	34809
**Nu**_**av**_	6.4724	6.4438	6.5492	6.5783	6.5782

### 3.3 Code validation

**Fig. 4:**
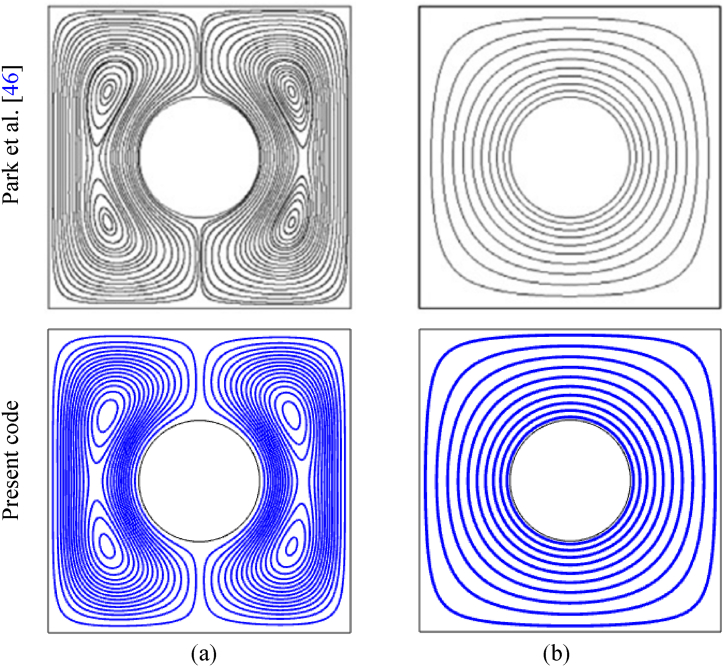
Outcomes of Park et al. [46]: (a) streamline and (b) isotherm contours.

The simulation's scientific validity is confirmed using the results of Park et al. [46], with *Ra* = 10^3^ and Pr = 0.7, where the circle's side length and radius are assumed to be 1 and 0.2, respectively. All of the outer walls acted as a cold surface, while the central circular surface provided a uniform heated wall (T_h_). This proposed work and Park et al. [46] both have a hot circular wall at the middle point and the rest of the walls were insulated. In Fig. 4, it is clear that the shapes of streamline and isotherm closely match the present results. Table 4 compares Nu_av_ of the inner circular hot wall to Park et al.'s [46] findings, which show a similar outcome, where the absolute errors are very low. These results are consistent with the current study, boosting confidence in the analysis.

**Table 4:** Association of Nu_av_ (of circular hot wall) between the present study with [46].

Ra	Park et al. [46]	Current Study	Difference
10^3^	5.024	5.023	0.1 %
10^4^	5.129	5.132	0.3 %
10^5^	7.817	7.816	0.1 %
10^6^	14.298	14.296	0.2 %

## 4 Results and discussion

The impact of magnetic field on natural convection within a trapezoidal cavity containing *Ag-SiO*_*2*_*-H*_*2*_*O* hybrid nanofluid is investigated using streamlines, isotherms, line graphs, and response surfaces for changing values of *Ra*, *Ha*, and *ϕ*. To further demonstrate how *Ra*, *Ha,* and *ϕ* impact on response function (Nu_av_), sensitivity analysis is carried out. While water (*H*_*2*_*O*) is classified as a basic fluid, silver (*Ag*) and silicon dioxide (*SiO*_*2*_) nanoparticles are thought to be spherical. In these numerical studies, several important factors are employed as fixed values, such as Pr = 6.83, *Ra* = 10^4^, *Ha* = 10, *ϕ*_*Ag*_ = 0.02, and *ϕ*_*SiO*2_ = 0.02.

### 4.1 Effects of Rayleigh number

**Fig. 5:**
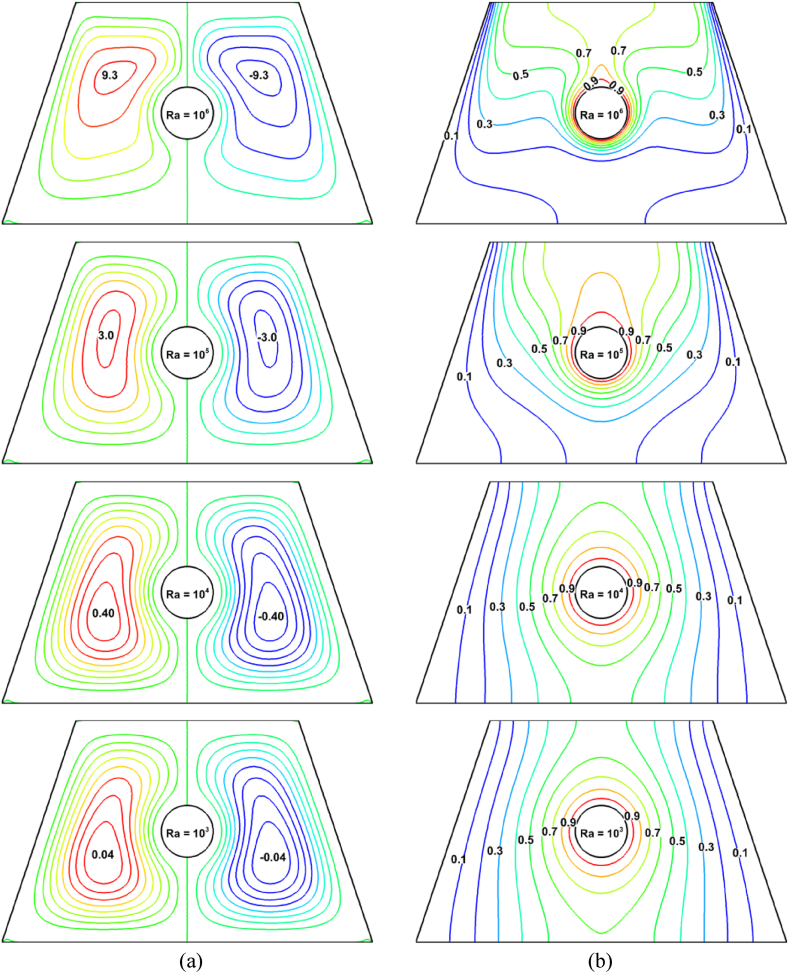
Consequence of *Ra* using (a) streamlines and (b) isotherms.

The impact of varying *Ra* magnitudes (10^3^ to 10^6^) on streamlines and isotherms is shown in Fig. 5, whereas *Ha* = 10, *ϕ*_*Ag*_ = 0.02, *ϕ*_*SiO2*_ = 0.02, and Pr = 6.83 stays constant at that point. Fig. 5(a) illustrates that for every value of *Ra*, two symmetric vorticities form in this trapezoidal cavity. While the right vorticity rotates in a clockwise trend, the left vorticity rotates counterclockwise.

**Fig. 6:**
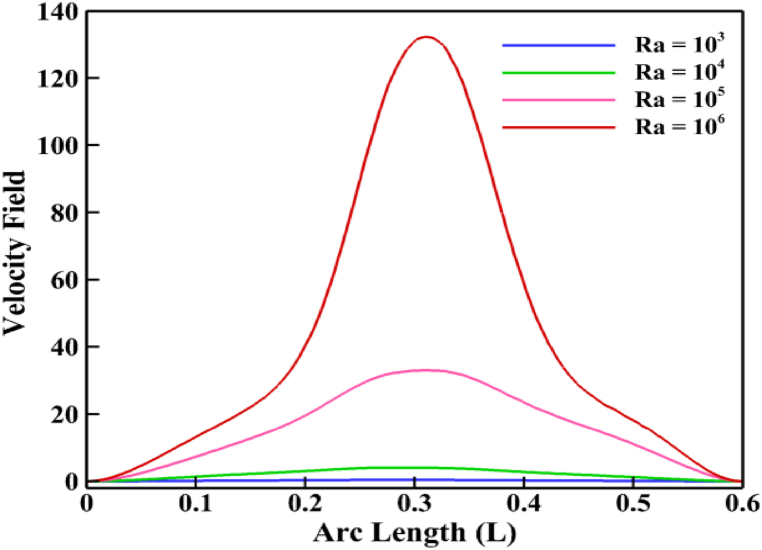
Velocity profile for *Ra*.

After entering the low-temperature region, the heavy liquid spins in place as it presses up against the bottom adiabatic wall of the thermal boundary layer. The line graph in Fig. 6 from (0.39, 0) to (0.39, 0.6) shows that the fluid flow in this cavity increases as Rayleigh's number increases. This suggests that a physical explanation for fluid flow is that the buoyancy effect increases the natural convective tendency as *Ra* increases. A very slight change in the flow rate occurs as *Ra* moves from 10^3^ to 10^4^. However, with larger increases in *Ra*, the fluid flows increase significantly, as shown in the streamlines for *Ra* values 10^5^ and 10^6^. The biggest changes in streamline are observed at *Ra* = 10^6^, where a larger vortex is found. Furthermore, the physical interpretation is that *Ra* is a measure of the action of the buoyancy force, which raises the streamline flow as *Ra* rises. On the other hand, isotherm contours provide an explanation of the heat transmission mechanism. The effect of *Ra* on the isotherm contours inside the trapezoidal arena is seen in Fig. 5(b). The isotherm contours along the heated surface and cool wall are almost parallel when *Ra* values are low (10^3^ and 10^4^). This time, the heat transmission rate is 2.3 %. This implies that the Nu_av_ is rather insignificant for tiny values of *Ra*. Therefore, it is clear that when *Ra* increases, the isotherm contour lines distort. The rate of Nu_av_ develops up to 59 % as a result of *Ra* changing from 10^4^ to 10^5^. At the highest levels of *Ra* (10^5^ and 10^6^), this phenomenon is very noticeable, and indicating that the rate of heat transfer is sufficiently larger at high *Ra* than it is at low *Ra*.

### 4.2 Effect of Hartmann number

**Fig. 7:**
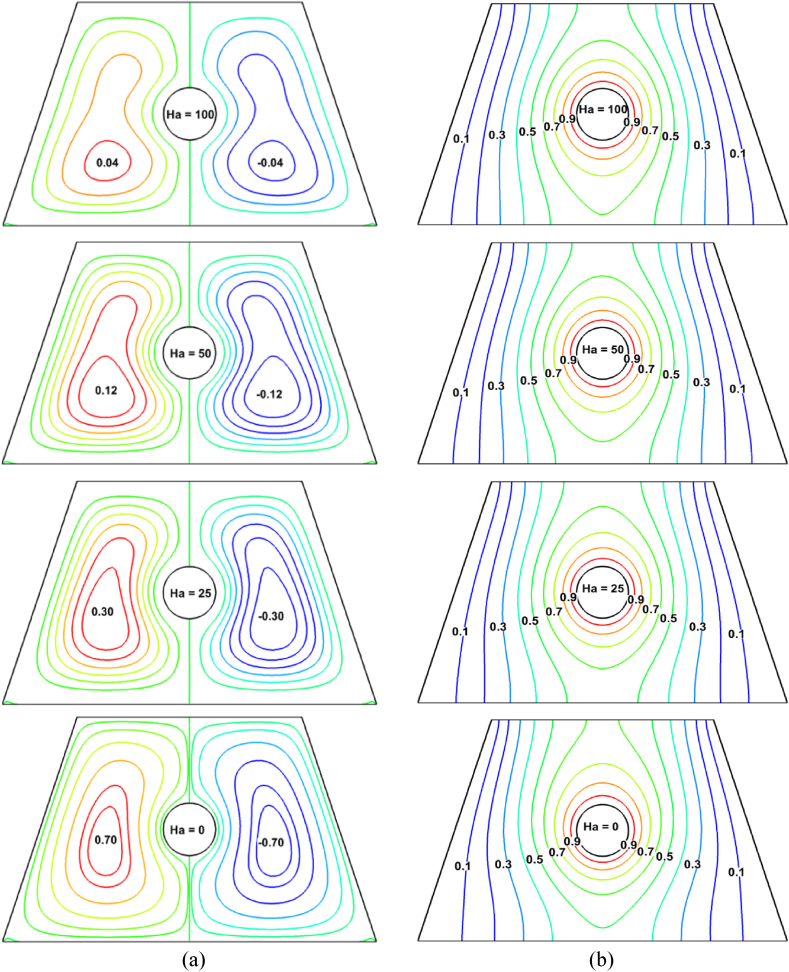
Consequence of *Ha* using (a) streamlines and (b) isotherms.

**Fig. 8:**
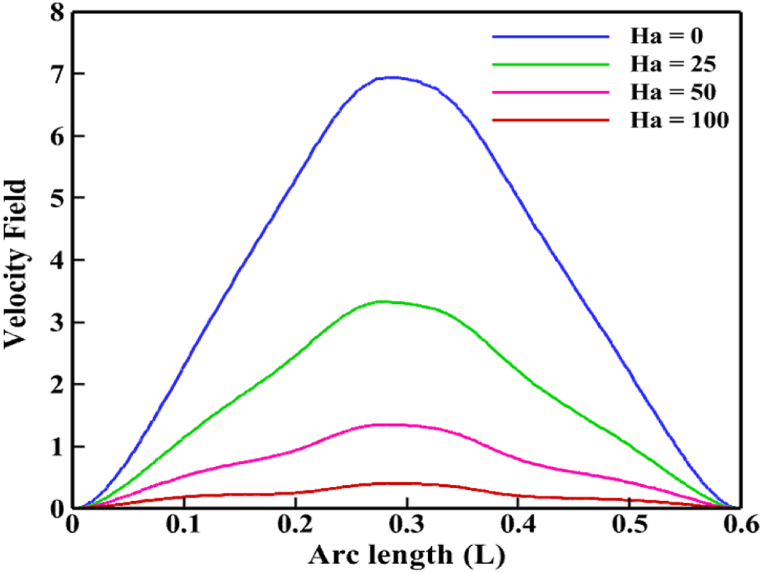
Velocity profile of *Ha*.

The effects of the hydromagnetic field, as shown by the Hartman number (*Ha*), on streamlines, isotherms, and velocity profiles are described in Fig. 7, Fig. 8 by maintaining *Ra* = 10^4^, *ϕ*_*Ag*_ = 0.02, *ϕ*_*SiO2*_ = 0.02, and Pr = 6.83. Fig. 7(a) displays the streamline fluctuation for various *Ha* magnitudes. For all values of *Ha*, it appears that two symmetric rotating cells form inside the cavity on either side of the heated surface. While the right cell is facing clockwise, the left cell is oriented counterclockwise ward. The rotational strength gradually decreases as the *Ha* magnitude increases. The increasing impact of the magnetic field results from the development of *Ha*, which generated the Lorentz force. The fluid movement within the cavity is directly opposed by these Lorentz forces, which are at their strongest when *Ha* = 100. This is *Ha*'s primary physical effect. As a result, the level of vortices is lower than those where *Ha* = 0.

In other words, a line graph in Fig. 8 that follows the line (0.39, 0) to (0.39, 0.6) shows that the flow inside the enclosure somewhat decreases as the *Ha* increases. Additionally, when *Ha* is missing, the isotherms show the maximum heat flow rate, as seen by the isothermal lines in Fig. 7(b). The density and isotherm lines eventually become less flat as *Ha* rises. The rate of heat transmission decreases by up to 2.4 % when the magnetic field is applied (*Ha* rises to 25). Even if the impacts of the adjustments on isotherm lines are minimal, streamlines also have a minor impact. In reality, the applied magnetic field is what creates the obstacle to fluid movement. In other words, when *Ha* = 100, the conventional heat transfer rate is relatively low (Nu_av_ = 6.8) when the magnetic effect is maximum. The rate of heat transmission will also be lower due to the slight decrease in fluid connection brought on by the stronger magnetic field. The Nu_av_ is decreased by 3.12 % as a result of the highest magnetic field effect.

### 4.3 Importance of hybrid nanofluid

**Fig. 9:**
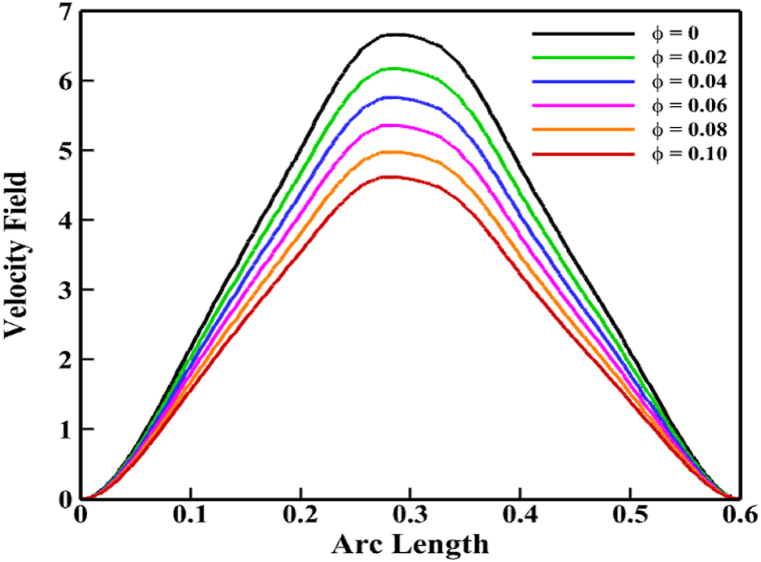
Velocity profile of *ϕ* along a line (0.39, 0) to (0.39, 0.6).

**Fig. 10:**
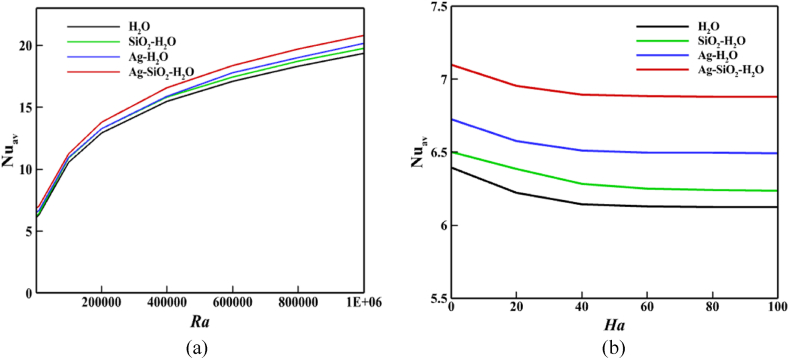
Judgment of thermal playacting by Nu_av_ for: (a) *Ra*, and (b) *Ha*.

Fig. 9, Fig. 10 explains the impacts and importance of adding solid nanoparticles to base fluid. The total fluid velocity is somewhat decreased as a result of the solid nanoparticle buildup. Due to the fact that in the fluid domain, these solid particles show some resistance to fluid motion. Consequently, *ϕ* decreases its velocity, as Fig. 9 makes evident. When solid nanoparticles are present throughout the domain, fluid molecules begin pressing against one another, which lowers fluid velocity. For fluid flow, this is the primary physical explanation of *ϕ*. But the Nu_av_ has greatly improved as a result of the escalation of *ϕ*. A line graph is used in Fig. 10(a) and (b) to illustrate the importance of solid nanoparticle formation in water. Here, the Nu_av_ is utilized to explain and demonstrate the rate of heat transmission for various fluid types using the possessions of *Ra* and *Ha*. For pure fluid (*H*_*2*_*O*), *SiO*_*2*_*-H*_*2*_*O* nanofluid, *Ag-H*_*2*_*O* nanofluid, and *Ag-SiO*_*2*_*-H*_*2*_*O* hybrid nanofluid, Fig. 10(a) shows that the Nu_av_ grows monotonically with the increases of *Ra*. Without the existence of any nanoparticles in water, the rate of Nu_av_ is 6.3234 by taking all others in standard form. When $\phi_{SiO_{2}}=0.02$ and *ϕ*_*Ag*_ = 0, the addition of *SiO*_*2*_ nanoparticles quickly improves the Nu_av_ (6.4625) relative to the pure base fluid which is 2.1 % greater than the base fluid. A similar manner exists for adding Ag nanoparticles ($\phi_{SiO_{2}}=0$ and *ϕ*_*Ag*_ = 0.02) to improve the Nu_av_ (6.6610) relative to the pure base fluid which is 5.3 % superior to the base fluid. But both of the solid nanoparticles ($\phi_{SiO_{2}}=0.02$ and *ϕ*_*Ag*_ = 0.02) increased the Nu_av_ significantly which is 7.0379. This time, the improvement rate is 11.29 %.

In addition, Fig. 10(b) illustrates how adding mono or hybrid nanoparticles enhances Nu_av_ for distinct values of *Ha*. The findings indicate that the *Ag-SiO*_*2*_*-H*_*2*_*O* hybrid nanofluid has a larger Nu_av_ than *Ag-H*_*2*_*O* nanofluids, yet for all fluid combinations, the rate of Nu_av_ decreases as the *Ha* factor rises. This indicates that because of the hybrid nanofluid's exceptional thermal properties, Fig. 10 shows that the *Ag-SiO*_*2*_*-H*_*2*_*O* hybrid nanofluid performs better in terms of heat transport than either *SiO*_*2*_*-H*_*2*_*O* or *Ag-H*_*2*_*O* nanofluid. The primary goal for utilizing hybrid nanofluid as opposed to base fluid or nanofluid containing a particular nanoparticle is this.

### 4.4 Response surface methodology

The impact of the included parameters (*Ra*, *Ha,* and *ϕ*) on Nu_av_, is the response function, for this fluid model is described by using a popular statistical procedure called response surface methodology (RSM). One effective method for illustrating multivariate scenarios where the input factors concurrently influence the attention-grabbing replies is RSM [47,48]. The second-order RSM model typically provides an acceptable estimate of the response, despite the existence of other RSM models. The quadratic RSM model is considered as:

(12) $$y=d_{0}+\sum_{i=1}^{3}d_{i}x_{i}+\sum_{i=1}^{3}d_{ij}x_{i}x_{j}+\sum_{i=1}^{3}d_{ii}x_{i}^{2}$$

Here, *y* is the output function, $d_{0},d_{i},d_{ij}\;\text{and}\;d_{ii}$ are the coefficients of the respective terms. Furthermore, Nu_av_ is measured as the response faction (*y*), while the pertinent factors *Ra*, *Ha,* and *ϕ* are considered as contribution parameters. Finding the best-fitted connection among between Nu_av_ and independent factors is the major goal for using RSM. In this instance, a second-order RSM model based on central composite design (CCD) is used [49]. Twenty (20) runs comprise this RSM model based on CCD (8 cubes, 6 centers, and 6 axial points). The codded level for CCD-based RSM is given in Table 5. Also, the magnitude of the response function for 20 runs with codded and actual levels is described in Table 6.

**Table 5:** Actual and codded values for CCD based RSM.

Factors	Codded and Actual Level		
	Lowest value (−1)	Medium value (0)	Highest value (1)
*Ra*	10^3^	500500	10^6^
*Ha*	0	50	100
*ϕ*	0	0.05	0.1

**Table 6:** Distinct arrangement of input factors with response function.

Run Order	Codded Values			Real Values			Response
	*Ra*	*Ha*	*ϕ*	*Ra*	*Ha*	*ϕ*	Nu_av_
1	0	0	0	500500	50	0.05	12.867
2	−1	0	0	1000	50	0.05	7.0775
3	0	0	0	500500	50	0.05	12.867
4	0	−1	0	500500	0	0.05	18.218
5	0	1	0	500500	100	0.05	8.6668
6	0	0	1	500500	50	0.1	13.535
7	1	1	−1	1000000	100	0	10.88
8	0	0	0	500500	50	0.05	12.867
9	−1	1	1	1000	100	0.1	8.1404
10	−1	−1	−1	1000	0	0	6.1228
11	1	−1	1	1000000	0	0.1	23.337
12	0	0	0	500500	50	0.05	12.867
13	−1	−1	1	1000	0	0.1	8.142
14	0	0	0	500500	50	0.05	12.867
15	−1	1	−1	1000	100	0	6.1198
16	0	0	−1	500500	50	0	12.357
17	1	−1	−1	1000000	0	0	19.576
18	0	0	0	500500	50	0.05	12.867
19	1	0	0	1000000	50	0.05	16.632
20	1	1	1	1000000	100	0.1	11.634

Moreover, in Table 7, the conclusions of this RSM-based statistical analysis are described using analysis of variance (ANOVA). The extreme number of self-governing terms is indicated by the degrees of freedom or DOF. Furthermore, a crucial pointer of this analysis is the p-value, which expresses the possibility that the null hypothesis would hold true for a particular statistical model. An extremely small p-value (often fewer than 5 %) specifies that the archetypal is significant since a low p-value implies the rejection of the null hypothesis. The statistical assessment of this framework and the testing approaches indicate that the R^2^ (97.17 %) for Nu_av_ is better demonstrating that this design is apposite for computing the Nu_av_. Despite having a much smaller adjusted-R^2^ (94.63 %) than the R^2^ (97.17 %) of Nu_av_, the model is still able to accurately replicate the experimental results. Lack-of-fit is another critical metric that must be extremely small for a model to be accepted as adequate. RSM developed a general model, indicated below, to investigate the connection between Nu_av_ and the parameters *Ra*, *Ha,* and *ϕ*:

(13) $$y=d_{0}+d_{1}Ra+d_{2}\;Ha+d_{3}\;\phi +d_{12}Ra.Ha+d_{13}Ra.\phi +d_{23}\;Ha.\phi +d_{11}Ra^{2}+d_{22}\;Ha^{2}+d_{33}\;\phi^{2}$$

where $d_{0},d_{1},d_{2},d_{3},d_{11},d_{22},d_{33},d_{12},d_{13}$ and $d_{23}$ indicate the corresponding coefficients of the regression line generated by this RSM for the factors *Ra*, *Ha,* and *ϕ*.

**Table 7:** Outcomes of ANOVA for Nu_av_.

Source	DOF	F-Value	p-Value	Comment
**Model**	9	38.18	<0.0001	**Significant**
*Ra*	1	210.44	<0.0001	
*Ha*	1	69.67	<0.0001	
*ϕ*	1	9.65	0.0111	
*Ra*^*2*^	1	7.59	0.0203	
*Ha*^*2*^	1	0.0059	**0.9405**	
*ϕ*^*2*^	1	0.0317	**0.8623**	
*Ra.Ha*	1	37.64	0.0001	
*Ra.ϕ*	1	0.3335	**0.5764**	
*Ha.ϕ*	1	0.5012	**0.4952**	
**Lack-of-Fit**	5	–	–	**Insignificant**

∗∗Here, **R**^**2**^ = **97.17 %, Adjusted R**^**2**^ = **94.63 %**.

Furthermore, Nu_av_'s predicted coefficients of equation (13) are shown in Table 8 and are computed as coded units. An acceptable regression equation has only been constructed using the significant terms which have small p-values due to their significance. Conversely, the terms that are not significant have been ignored (bold emphasized).

**Table 8:** Coefficients of regression line obtained from RSM.

Coefficients	$d_{0}$	$d_{1}$	$d_{2}$	$d_{3}$	$d_{11}$	$d_{22}$	$d_{33}$	$d_{12}$	$d_{13}$	$d_{23}$
Values	3.6	0.6728	−0.3871	0.1441	−0.2436	−0.0068	−0.0157	−0.3181	−0.0299	−0.0367
p-values	–	<0.0001	<0.0001	0.0111	0.0203	**0.9405**	**0.8623**	0.0001	**0.5764**	**0.4952**

That is, the term *Ha*^*2*^, *ϕ*^*2*^, *Ra*.*ϕ,* and *Ha.ϕ* are entirely insignificant for the following regression line denoted by equation (13). Therefore, the following mathematical summary can be used to describe the interconnection between Nu_av_ and the factors *Ra*, *Ha,* and *ϕ*:

(14) $${\text{Nu}}_{\text{av}}=3.6+0.6728Ra-0.3871Ha+0.1441\;\phi -0.2436Ra^{2}-0.3181Ra.Ha$$

### 4.5 Response surface analysis

**Fig. 11:**
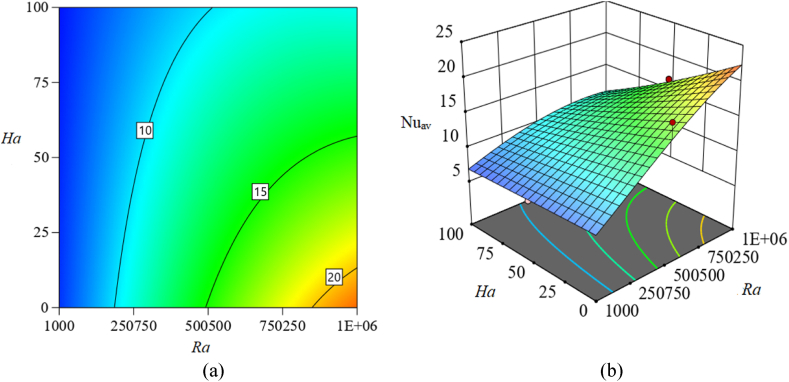
Consequence of *Ra* and *Ha* on Nu_av_: (a) 2*D* view; (b) 3*D* view.

**Fig. 12:**
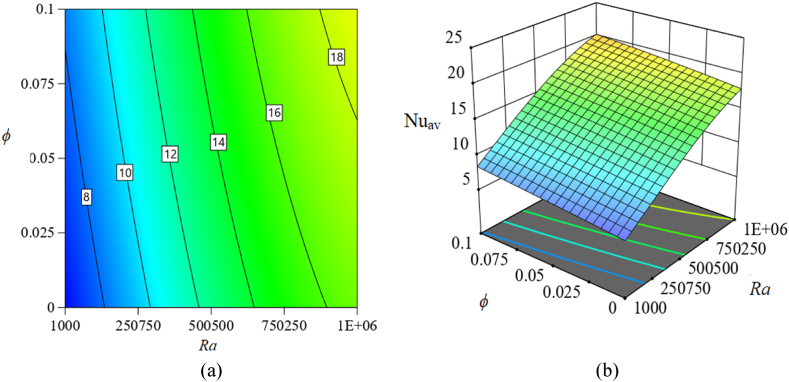
Effect of *Ra* and *ϕ* on Nu_av_: (a) 2*D* view; (b) 3*D* view.

**Fig. 13:**
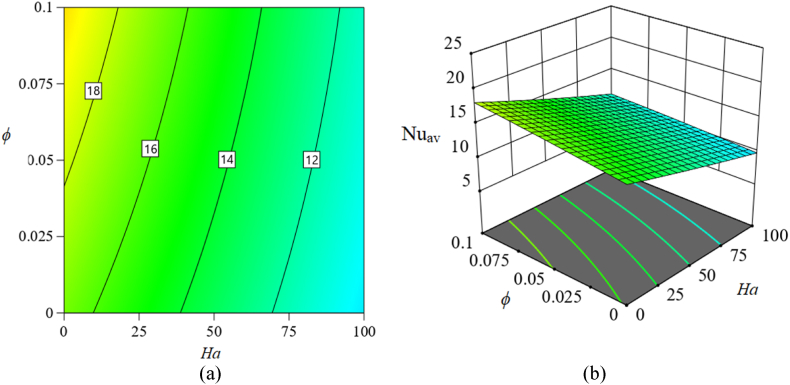
Consequence of *Ha* and *ϕ* on Nu_av_: (a) 2*D* view; (b) 3*D* view.

Fig. 11, Fig. 12, Fig. 13 in this part show 2*D* and 3*D* contour plots for response surfaces achieved from RSM to explore the control of autonomous parameters on the Nu_av_. The control of *Ra* and *Ha* on Nu_av_ is described in Fig. 11. The magnitude of the response function is increased which is apparent from the two-dimensional contour plot in Fig. 11(a). In a certain magnitude of the magnetic field, the rate of Nu_av_ is advanced while *ϕ* is taken perpetually. For instance, for raising the magnitude of *Ra* to 500500 from 10^3^, the heat transfer rate (Nu_av_) is enlarged by about 300 %. Also, for changing *Ra* = 10^6^ from 500500, this rate is improved by 18.3 %. In addition, Fig. 11(b) illustrates a 3-dimensional view for perceiving the consequences of *Ra* and *Ha* on Nu_av_. Furthermore, Additionally, Fig. 12 illustrates additional 2*D* and 3*D* visual representations to represent the influence of *Ra* and *ϕ* on Nu_av_. Additionally, the increase in *Ra* and *ϕ* raises the rate of Nu_av_ while keeping *Ha* constant. This changing rate of Nu_av_ is dominant at the highest value of *Ra* and *ϕ*, as the 2*D* contour Fig. 12(a)–(b) makes evident. Likewise, Fig. 13 represents the influence of *Ha* and *ϕ* on Nu_av_. Here, as the magnetic field (*Ha*) decreases, the size of *ϕ* increases, developing the changing rate of Nu_av_. However, compared to the last two examples, Nu_av_'s change rate is lower.

### 4.6 Sensitivity analysis

Sensitivity analysis, which ascertains how uncertainties in a model's input affect the response, is a basic part of numerical simulation. To assess the extent to which the model's parameters impact the result variables, one possible meaning of the phrase is to conduct a "sensitivity analysis" [50]. The most important parameters can be arranged according to their level of importance using the sensitivity analysis results to determine which parameter performs the best. The resultant variables' sensitivity to the substantial input factors (*Ra*, *Ha,* and *ϕ*) is found to exhaust the partial derivatives of Nu_av_ about independent components. This results in the response function Nu_av_ being constructed, and equation (14) for the input parameters is as follows:

(15) $$\frac{\partial {\text{Nu}}_{av}}{\partial Ra}=0.6728-0.4872Ra-0.3181Ha$$

(16) $$\frac{\partial {\text{Nu}}_{av}}{\partial Ha}=-0.3871-0.3181Ra$$

(17) $$\frac{\partial {\text{Nu}}_{av}}{\partial \phi }=0.1441$$

**Fig. 14:**
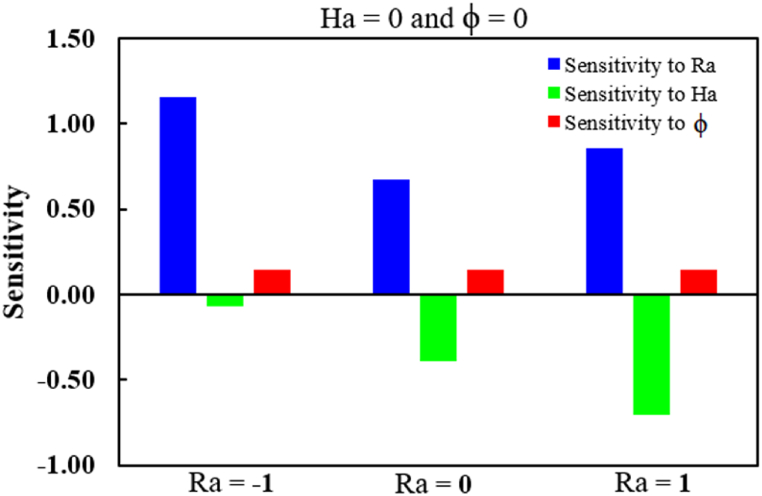
Sensitivity of Nu_av_ at *Ra* = 0 and *ϕ* = 0.

The rate at which the Nu_av_ is sensitive to *Ra*, *Ha,* and *ϕ* can be determined using equations (15), (16), (17). The data concerning sensitivity are displayed in Table 9. Using this model, the following quantities are obtained: *ϕ* at levels 0, -1, and 1 (0, 50 and 100), *Ra* at levels 0 and 1 (500500 and 106), and *Ha* at levels −1, 0, and 1 (0, 50 and 100). Moreover, a positive sensitivity indicates that the reaction is increased by the input parameters. This demonstrates that the Nu_av_ benefit from *Ra* and *ϕ*. Conversely, a negative sensitivity shows exactly the opposite trend, whereby raising the input parameters results in a reduction in the reaction. The bar diagram in Fig. 14 indicates that *Ra* and *ϕ* have positive sensitivity, but *Ha* has negative sensitivity, where the bar length expresses the level of sensitivity. That is, the parameter Ra has the highest sensitivity rather than *Ha* and *ϕ*.

**Table 9:** Sensitivity analysis for Nu_av._

*Ra*	*ϕ*	*Ha*	$\frac{\partial {\text{Nu}}_{av}}{\partial Ra}$	$\frac{\partial {\text{Nu}}_{av}}{\partial Ha}$	$\frac{\partial {\text{Nu}}_{av}}{\partial \phi }$
−1	0	−1	1.4781	−0.069	0.1441
		0	1.16	−0.069	0.1441
0		−1	0.9909	−0.3871	0.1441
		0	0.6728	−0.3871	0.1441
1		−1	0.5037	−0.7052	0.1441
		0	0.1856	−0.7052	0.1441
−1	1	−1	1.4781	−0.069	0.1441
		0	1.16	−0.069	0.1441
0		−1	0.9909	−0.3871	0.1441
		0	0.6728	−0.3871	0.1441
1		−1	0.5037	−0.7052	0.1441
		0	0.1856	−0.7052	0.1441

## 5 Conclusions

This study investigates the comportment of natural convective heat transformation in a confined trapezoidal enclosure using a hybrid (*Ag-SiO*_*2*_*-H*_*2*_*O*) nanofluid, with a special focus on how heat transport processes interact with the effects of magnetic fields. To represent the complex behavior, a numerical solution is created using the Galerkin finite element method. To conduct a complete assessment of the heat transfer mechanism, the sensitivity analysis of the response function is investigated using the statistical approach known as RSM. The consequence of hybrid nanofluid on Nu_av_, streamlines, and isotherms is investigated. Furthermore, surface plots in two and three dimensions provide a clear visual picture of the heat transfer process for key elements. The most important findings from numerical and statistical analysis are given below:

• Owing to the hybrid nanofluid's outstanding thermal properties, *Ag-SiO*_*2*_*-H*_*2*_*O* outperforms base fluid and mono-nanofluid in terms of heat transmission, increasing it by 11.29 % and 8.9 %, respectively.
• The velocity and temperature distribution are adversely affected by the *Ha* right away. Also when a magnetic field is applied (*Ha* = 25), the rate of heat transmission drops by up to 2.4 %.
• The sensitivity analysis findings suggest that *Ha* is unfavorably sensitive to heat transference, while *Ra* and *ϕ* demonstrate positive sensitivity to Nu_av_.

**Limitations and Future Efforts:** To analyze the fluid flow, heat, and mass transfer for a 3*D* complex-shaped geometry, it is difficult to compute numerical solutions with a low-configured computer. Also, heat transmission and fluid flow processes in a closed cavity can be studied in depth. There are several ways to expand this study in the future:

• Multiple external effects can be considered including ternary nanoparticles, mass transpiration, thermal radiation, etc.
• Different non-Newtonian fluids can be analyzed as similar to numerous artificial neural network (ANN, CNN, etc.) based techniques.

## CRediT authorship contribution statement

**Sweety Khatun:** Writing – original draft, Methodology, Formal analysis, Data curation. **Rupa Kundu:** Writing – original draft, Data curation. **Saiful Islam:** Writing – review & editing, Supervision. **Ritu Aktary:** Writing – original draft, Resources, Investigation. **Dipankar Kumar:** Supervision.

## Funding

There was no external support for this study.

## Declaration of competing interest

On behalf of all authors, I declared that we have no known competing financial interests or personal relationships that could have appeared to influence the work reported in this paper.

## Figures and Tables

**Table:** 

*c*_*p*_	Specific heat (J.kg^−1^.K^−1^)	*α*	Thermal diffusivity (m^2^.s^−2^)
*g*	Acceleration for gravity (m.s^−1^)	*β*	Coefficient of thermal expansion (K^−1^)
*B*_*0*_	Magnetic field strength (N/Am^2^)	*ρ*	Density (kg.m^−3^)
L	Enclosure length (m)	*ϕ*	Particle concentration
H	Enclosure height (m)	*θ*	Non-dimensional temperature
*Ag*	Silver	*μ*	Dynamic viscosity (kg.m^−1^.s^−1^)
*SiO*_*2*_	Silicon dioxide	*ʋ*	Kinematic viscosity (m^2^.s^−1^)
*Ha*	Hartmann number	Ω	Vorticity vector
*Pr*	Prandtl number	*σ*	Electric conductivity (Ω^−1^.m^−1^)
*Ra*	Rayleigh number	*ψ*	Stream function
*Nu*	Nusselt number		
*p*	Dimensional pressure (N.m^−2^)	**Subscripts:**	
*P*	Dimensionless pressure	av	Average
t	Dimensional time (s)	bf	Base fluid
T	Dimensional temperature (K)	hnf	Hybrid nanofluid
*u*, *v*	Dimensional velocity component (m.s^−1^)	sp	Solid particle
*U*, *V*	Dimensionless velocity component		
*x*, *y*	Dimensional Cartesian coordinates		
*X*, *Y*	Dimensionless Cartesian coordinates		
